# Supplementary material for: Cognitive and Emotional Symptoms Induced by Chronic Stress Are Regulated by EGR1 in a Subpopulation of Hippocampal Pyramidal Neurons
Source: Int J Mol Sci. 2023 Feb 14;24(4):3833. doi: 10.3390/ijms24043833 (PMC9962724; doi:10.3390/ijms24043833)
Supplement: Supplementary file 1 [file ijms-24-03833-s001.zip › ijms-2183753-supplementary.pdf]

SUPPLEMENTARY MATERIAL

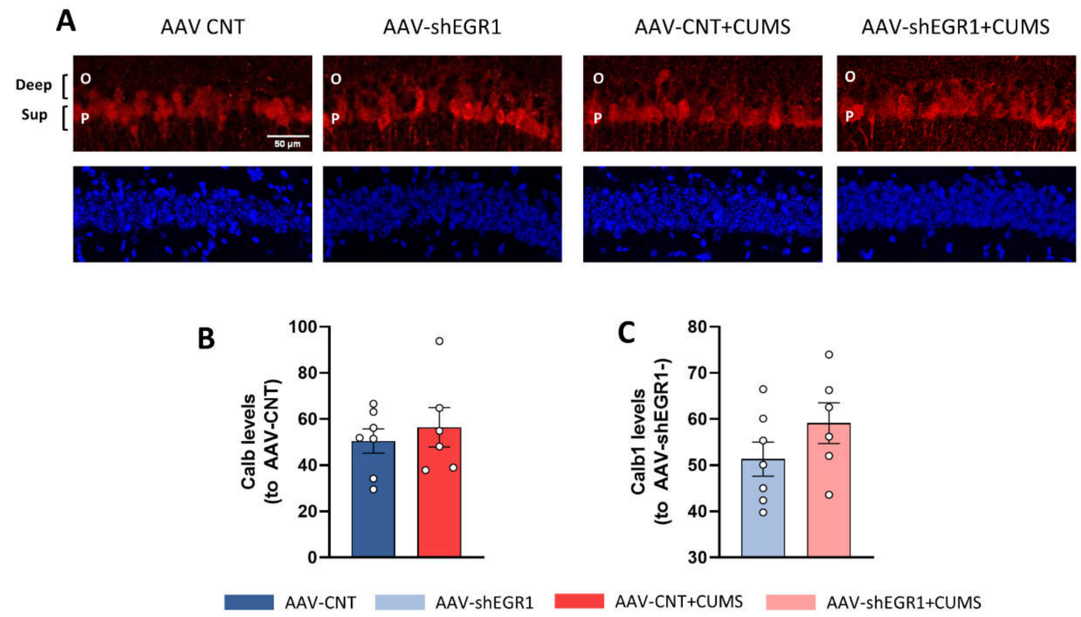

Supplementary Figure S1: Calb1 levels are not altered after Egr1 downregulation. Representative images of Calb1 staining in the CA1 of the hippocampus (**A**). Quantification of Calb1 optical density (IOD) in mice injected with AAV5-CAG-FLEX-GFP (**B**). Quantification of Calb1 optical density (IOD) in mice injected with AAV-shEGR1 (**C**). N=6-7 mice per group. O, Stratum oriens; P, Stratum pyramidale.
